# The complete chloroplast genome sequence of *Camellia sinensis* var. *sinensis* cultivar ‘FuDingDaBaiCha’

**DOI:** 10.1080/23802359.2022.2161327

**Published:** 2023-01-08

**Authors:** Dahe Qiao, Chun Yang, Yan Guo

**Affiliations:** Tea Research Institute, Guizhou Academy of Agricultural Sciences, Guiyang, China

**Keywords:** *Camellia sinensis*, chloroplast genome, FuDingDaBaiCha

## Abstract

The complete chloroplast (cp) genome sequence of *Camellia sinensis* var. *sinensis* cultivar ‘FuDingDaBaiCha’ (FD), one of the key contributors to the history of tea breeding in China, was determined in this study. The cp genome of FD is 157,025 bp in length, including a large single-copy (LSC, 86,586 bp), a small single-copy (SSC, 18,277 bp), and a pair of inverted repeats (IRa and IRb, 26,081 bp). The overall GC content is 37.3%. A total of 137 genes were predicted, including 92 protein-coding genes, 37 tRNA genes, and eight rRNA genes. Phylogenetic analysis showed that FD was closely related to *C. sinensis* cv. ‘AnHua’, *C. sinensis* cv. ‘QianCha 1’, and *C. sinensis* cv. ‘BanTianYao’. The determination of the complete cp genome sequence of FD provides a way for the subsequent study of the genetic background and phylogenetic relationships of different tea plant cultivars.

## Introduction

The tea plant (*Camellia sinensis* (L.) O. Kuntze 1881) is an evergreen woody plant belonging to *Camellia* genus of the Theaceae, native to China. Its leaves are the raw materials for the production of tea, one of the three major nonalcoholic beverages in the world (Brody 2019). *Camellia sinensis* var. *sinensis* cv. ‘FuDingDaBaiCha’ (FD) is one of the earliest clonal tea plant cultivars recognized at the national level in China (Wang et al. 2019). It is also the largest tea plant cultivar planted in China and even worldwide. Previous studies based on SSR and SNP markers from the nuclear genome revealed that FD might be the potential parent of many elite tea plant cultivars in China (Tan et al. 2015; Zhang et al. 2020), indicating that FD was one of the most widely used parents in tea plant breeding. However, due to the long breeding cycle and the loss or lack of accurate breeding records at an early stage, the genetic background of many tea plant cultivars is unclear or wrong. At the same time, due to the complexity of the genetic evolution of tea plants, it is not effective to identify genetic relationships using nuclear genome molecular markers alone (Niu et al. 2019; Wang et al. 2020). A recent study found that the chloroplast (cp) genome of *C. sinensis* cv. ‘XinYang 10’ was completely consistent with that of *C. sinensis* cv. ‘TieLuoHan’, suggesting that they may have been derived from the same female parent (Yan et al. 2021). However, the former is from the Henan Province and the latter from the Fujian Province, with no breeding records being available to show that the two cultivars were related. This demonstrates the importance of the cp genome in germplasm identification. In this study, the complete cp genome sequence of FD was characterized, which contributes to the understanding of the genetic background and phylogenetic relationships of different tea plant cultivars.

## Materials and methods

**Figure 1.**
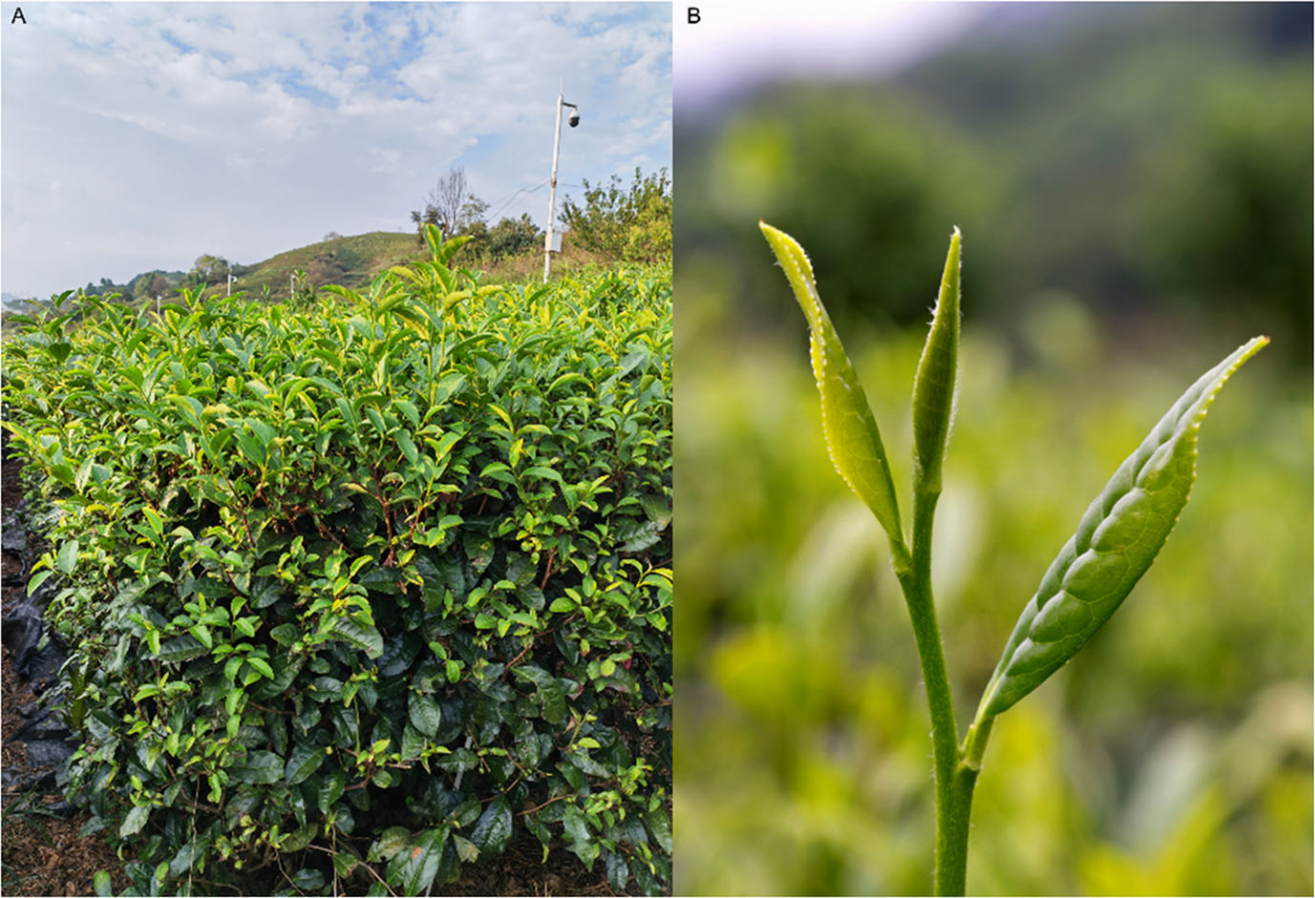
The species image of *C. sinensis* cv. ‘FuDingDaBaiCha’. (A) Whole plant morphology. (B) One bud and two leaves. The photos were taken by the authors in the Tea Germplasm Resource Nursery of Guizhou Province, Guiyang, Guizhou, China. More information on this cultivar can be found in the International Camellia Register (https://camellia.iflora.cn), under the registration ID ICR-22965.

Young leaves (the first leaf under apical bud) of FD were collected from a nine-year-old individual (Figure 1) planted in the Tea Germplasm Resource Nursery of Guizhou Province (N26°30′, E106°39′) in March 2021. Materials were collected following the rules and regulations of our institute, with no special permission statement being required for the collection. High-quality genomic DNA of FD was extracted from leaves using the Plant Genomic DNA Kit (TIANGEN, Beijing, China) according to the manufacturer’s instructions. A specimen was deposited in the Laboratory of Guizhou Tea Germplasm Innovation Engineering Technology Research Center at the Tea Research Institute, Guizhou Academy of Agricultural Sciences (http://www.gznkycys.cn/, Dr. Dahe Qiao, dahe10466@163.com) under the voucher number GZCS0001. Library construction and sequencing were performed by the Science Corporation of Gene (Guangzhou, China) on the Illumina Novaseq6000 platform based on the Paired-End 150 (PE150) strategy. The cp genome was *de novo* assembled using SPAdes v.3.5.0 (Lapidus et al. 2014) and annotated using CpGAVAS2 (Shi et al. 2019) and ORF Finder. The blastn and blastp methods were used to compare the preliminary annotated results with the reported proteins and rRNAs of the cp genomes of related species to verify the accuracy of the results. The circular structure of the cp genome was drawn using CPGView (http://www.1kmpg.cn/cpgview/).

## Results

**Figure 2.**
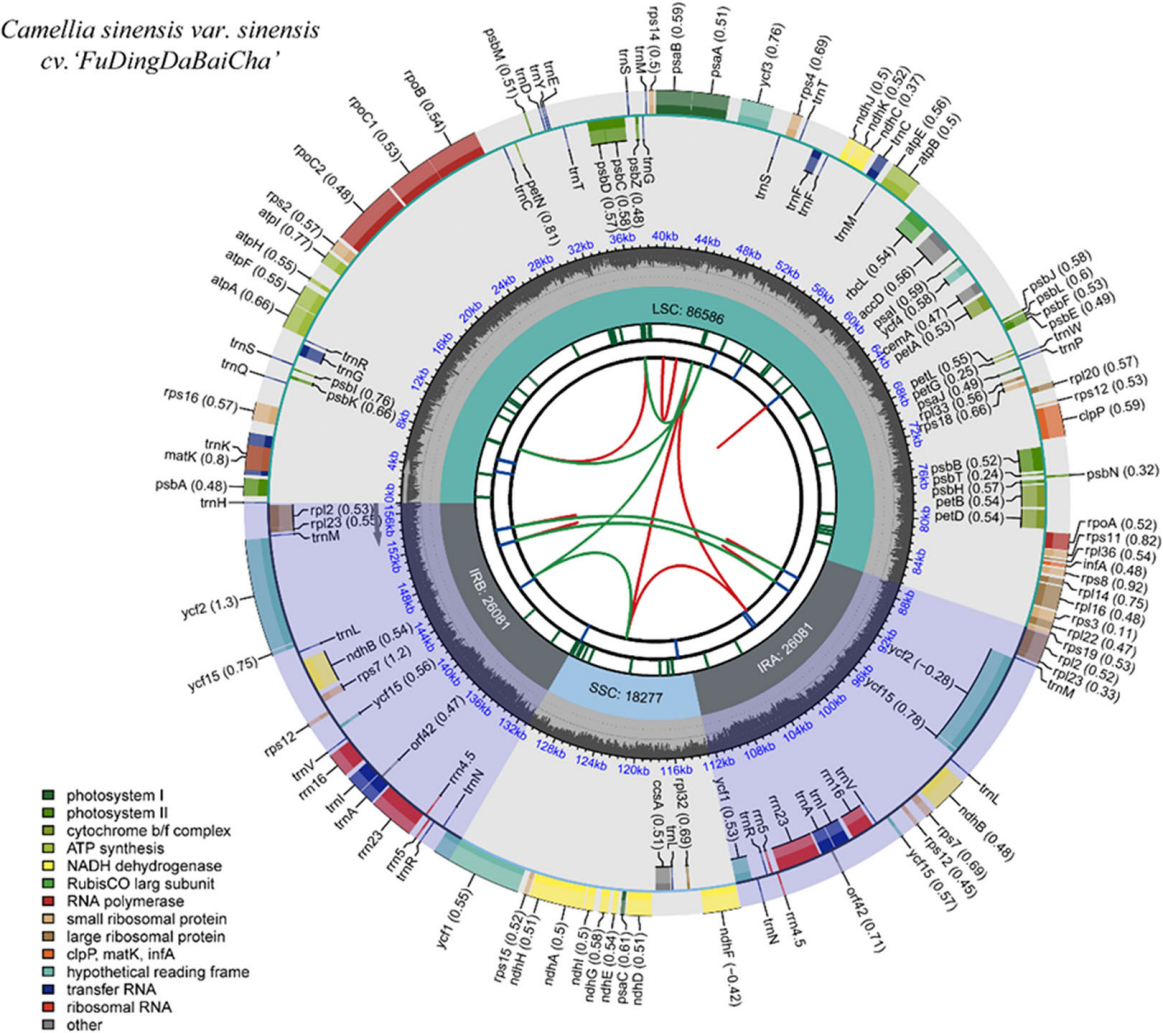
Schematic map of overall features of the chloroplast genome of *C. sinensis* cv. ‘FuDingDaBaiCha’. The map contains six tracks. From the center outward, the first track shows the dispersed repeats. The dispersed repeats consist of direct (D) and Palindromic (P) repeats, connected with red and green arcs. The second track shows the long tandem repeats as short blue bars. The third track shows the short tandem repeats or microsatellite sequences as short bars with different colors. The colors, the type of repeat they represent, and the description of the repeat types are as follows. Black: complex repeat; green: repeat unit size = 1; yellow: repeat unit size = 2; purple: repeat unit size = 3; blue: repeat unit size = 4; orange: repeat unit size = 5; red: repeat unit size = 6. The SSC, IRa, IRb, and LSC regions are shown on the fourth track. The GC content along the genome is plotted on the fifth track. The base frequency at each site along the genome is shown between the fourth and fifth tracks. The genes are shown on the sixth track. The optional codon usage bias is displayed in the parenthesis after the gene name. Genes are color-coded by their functional classification. The transcription directions for the inner and outer genes are clockwise and anticlockwise, respectively. The functional classification of the genes is shown in the bottom left corner.

A total of 108,157 paired-end clean reads isolated from the total DNA sequencing data were used for the cp genome assembly, and the cp genome of FD was obtained with the average coverage (X) of 207 (Tables S1 and S2; Figure S1). The complete cp genome of FD is 157,025 bp in length with a typical quadripartite structure (Figure 2). It contains a pair of inverted repeats (IRa and IRb) of 26,081 bp each, which are separated by a large single-copy (LSC, 86,586 bp) and a small single-copy (SSC, 18,277 bp). GC content of the two IRs, LSC, and SSC are 42.95%, 35.33%, and 30.55%, respectively. The overall GC content of the complete cp genome is 37.3%. A total of 137 genes were predicted, including 92 protein-coding genes, 37 tRNA genes, and eight rRNA genes. Among them, 13 cis-splicing genes including *rps16*, *atpF*, *rpoC1*, *ycf3*, *clpP*, *petB*, *petD*, *rpl16*, *rpl2* (2), *ndhB* (2), *ndhA*, and a trans-splicing gene *rps12* were detected by CPGview (http://www.1kmpg.cn/cpgview/) (Figure S2). The complete cp genome sequences and annotations of FD were submitted to the NCBI GenBank under the accession number MZ817088.

**Figure 3.**
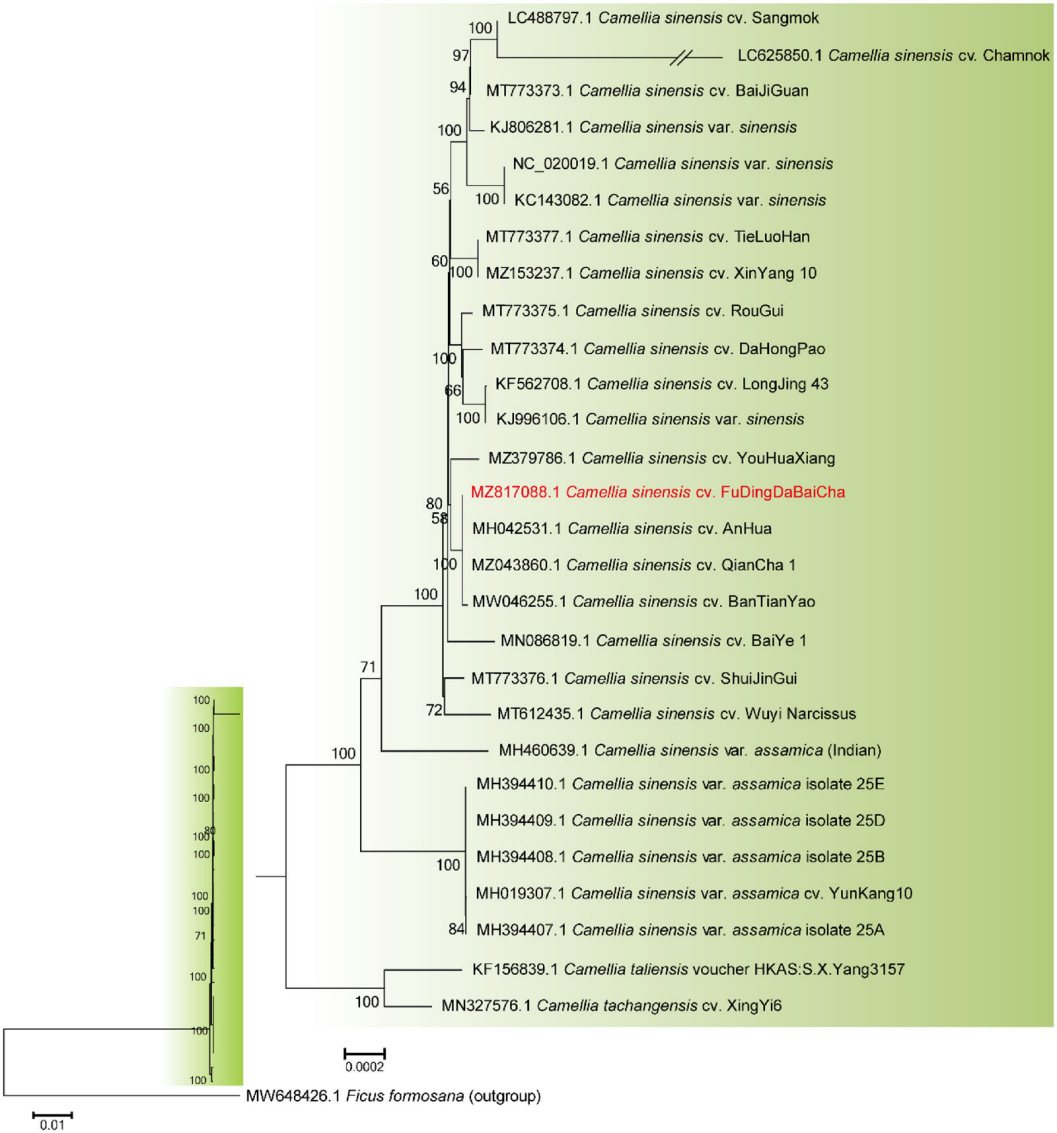
Phylogenetic relationships of different *Camellia sinensis* cultivars based on their complete chloroplast genome sequences. The phylogenetic tree was constructed using Neighbor-Joining (NJ) method with 1000 bootstrap replicates. The bootstrap values were labeled at each branch nodes. The branch of LC625850.1 was depicted as one fifth of the original branch length. The following published sequences were used to construct the NJ-tree: LC488797.1 (Lee et al. 2020), MT773373.1 (Fan et al. 2022), KJ806281.1 (Huang et al. 2014), MT773377.1 (Fan et al. 2022), MZ153237.1 (Yan et al. 2021), MT773375.1 (Fan et al. 2022), MT773374.1 (Li et al. 2021), KF562708.1 (Ye et al. 2014), MH042531.1 (Dong et al. 2018), MZ043860.1 (Yang et al. 2022), MW046255.1 (Fan et al. 2022), MN086819.1 (Hao, Wang, et al. 2019), MT773376.1 (Fan et al. 2022), MH019307.1 (Zhang et al. 2019), KF156839.1 (Yang et al. 2013), and MN327576.1 (Hao, Ma, et al. 2019).

To explore the phylogenetic position of FD, phylogenetic analysis was performed on the complete cp genomes of 28 tea plant cultivars with *Ficus formosana* as the outgroup species. All 29 complete cp genomes were aligned using the MAFFT v.7.475 software (Nakamura et al. 2018), and a Neighbor-Joining (NJ) tree was constructed using MEGA6 (Tamura et al. 2013) under the maximum composite likelihood model with 1000 bootstraps replicates (Figure 3). The results showed that FD was closely related to *C. sinensis* cv. ‘AnHua’ (Dong et al. 2018), *C. sinensis* cv. ‘QianCha 1’ (Yang et al. 2022), and *C. sinensis* cv. ‘BanTianYao’ (Fan et al. 2022), where the sequence of FD was identical to the former and differed by only one base from the latter two.

Considering that the cp genomes of more than 20 tea plant cultivars have been published, we further calculated their nucleotide diversity (Pi values) using the DnaSP 6 software (Rozas et al. 2017) to evaluate their sequence divergence levels. As shown in Figure S3, the Pi values of most sequences were below 0.005, indicating that the cp genomes of different tea plant cultivars were highly conserved. However, the Pi values in the SSC regions were much higher than that of other regions, indicating that SSC regions were the most divergent among different tea plant cultivars, which had the potential to develop molecular markers for population genetic studies. Then, we identified and analyzed simple sequence repeats (SSRs) in cp genomes of different tea plant cultivars based on MISA tools (Beier et al. 2017). A total of 64 (MH460639) to 71 (MF562708 and KJ996106) SSRs were identified in cp genomes of different tea plant cultivars, respectively. Tea plant cultivars with close genetic relationship had very similar SSR types and numbers (Figure S4). Based on these SSRs, we developed a marker that could be used to distinguish *C. sinensis* var. *sinensis* (CSS) and *C. sinensis* var. *assamica* (CSA) from China (Figure S5).

## Discussion and conclusions

*Camellia* is the largest genus in the family of Theaceae, containing more than 200 species (Wu et al. 2022). There is a huge variation in the length of the cp genome (153,044–157,353) for just the two major groups cultivated tea plant varieties (CSS and CSA). We also found that FD and ‘AnHua’ from Hunan (Dong et al. 2018) had identical cp genomes, indicating that they may have the same maternal parent. Additionally, we developed an SSR marker that can distinguish CSA and CSS, which provides a molecular basis for the identification of tea plant germplasm resources.

## Supplementary Material

Supplemental Material

## Data Availability

The data that support the findings of this study are openly available in GenBank of NCBI at https://www.ncbi.nlm.nih.gov/, under the accession no. MZ817088. The associated BioProject, SRA, and Bio-Sample numbers are PRJNA754486, SRR15458600, and SAMN20776252, respectively.
