# Supplementary material for: The complete chloroplast genome sequence of Camellia sinensis var. sinensis cultivar ‘FuDingDaBaiCha’
Source: Mitochondrial DNA B Resour. 2023 Jan 8;8(1):100–4. doi: 10.1080/23802359.2022.2161327 (PMC9833401; doi:10.1080/23802359.2022.2161327)
Supplement: Supplemental Material [file TMDN_A_2161327_SM4858.docx]

**Table S1.** Statistics and quality control of total DNA sequencing data in this study.

| **Sample** |  | **Length (bp)** | **Reads** | **Base (bp)** | **GC (%)** | **Q30 (%)** |
| --- | --- | --- | --- | --- | --- | --- |
| FD | Read1 | 150 | 4,430,192 | 664,528,800 | 38.83 | 92.65 |
|  | Read2 | 150 | 4,430,192 | 664,528,800 | 39.12 | 88.56 |

**Table S2.** Statistics and quality control of chloroplast genome sequencing data in this study.

| **Sample** |  | **Length (bp)** | **Reads** | **Base (bp)** | **GC (%)** | **Q30 (%)** | **Depth (X)** | **Coverage (%)** |
| --- | --- | --- | --- | --- | --- | --- | --- | --- |
| FD | Read1 | 150 | 108,157 | 16,223,550 | 36.53 | 93.36 | 207 | 100 |
|  | Read2 | 150 | 108,157 | 16,223,550 | 36.67 | 89.93 |  |  |


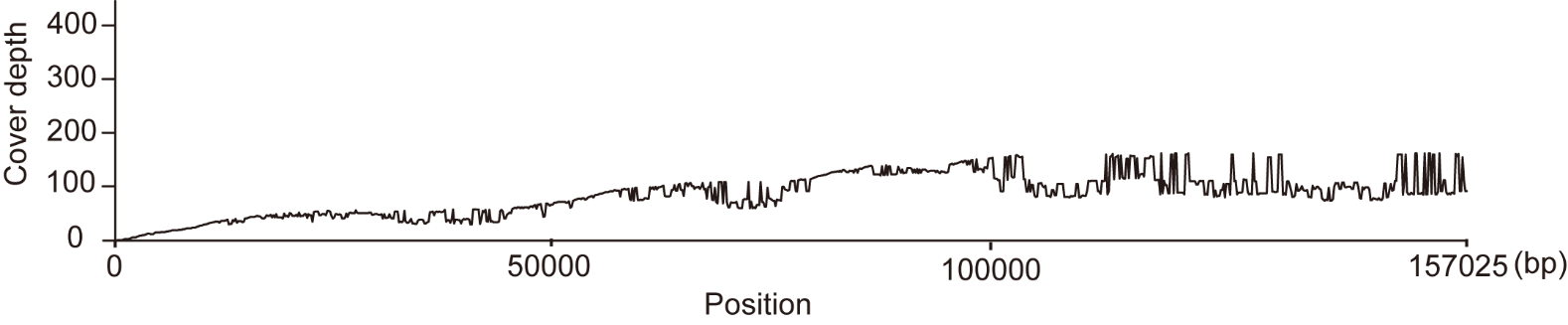


**Figure S1.** Quality assessment of FD chloroplast genome sequencing. The coverage depth of each base was calculated using bamdst (<https://github.com/shiquan/bamdst>).


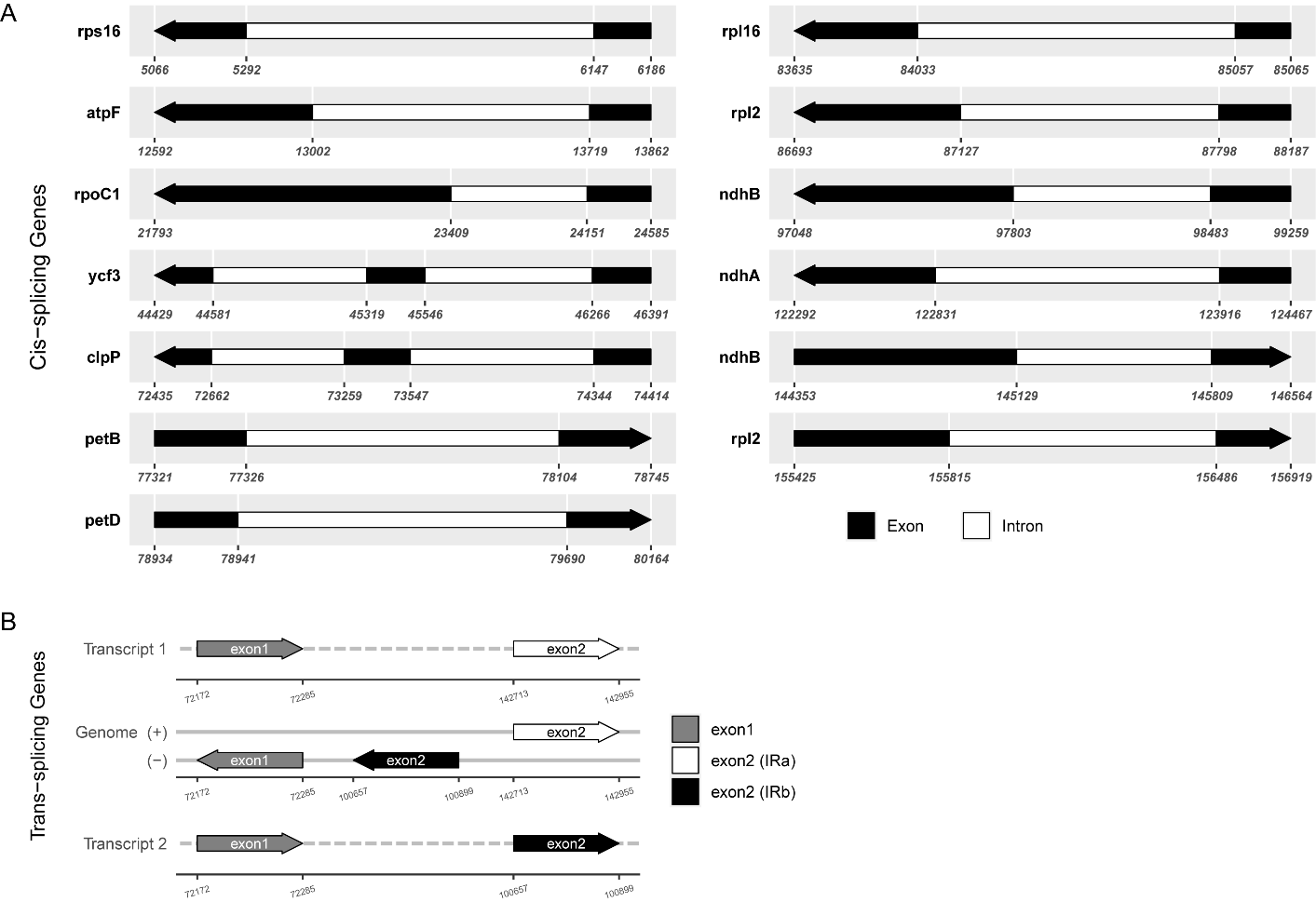


**Figure S2.** The splicing genes identified from the chloroplast genomes of *C. sinensis* cv. ‘FuDingDaBaiCha’ by using CPGview (http://www.1kmpg.cn/cpgview/). (A) Schematic map of the cis-splicing genes. The genes are arranged from top to bottom based on their order on the cp genome. The gene names are shown on the left, and the gene structures are on the right. The exons are shown in black and the introns are shown in white. The arrow indicates the sense direction of the gene. (B) Schematic map of the trans-splicing gene rps12. It has two unique exons. The exon2 is duplicated as it is located in the IR regions.


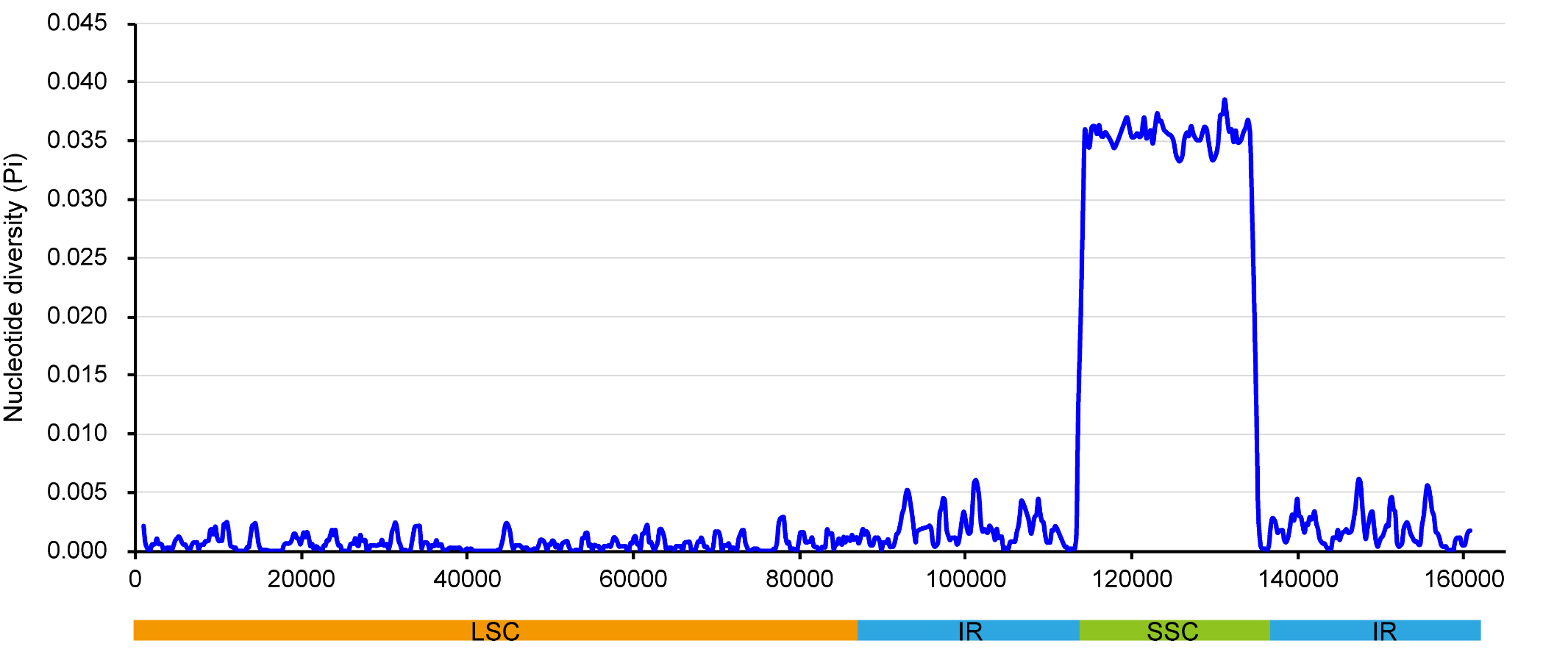


**Figure S3.** The nucleotide diversity of the cp genomes of different tea plant cultivars. The nucleotide diversity (Pi values) were calculated by DnaSP 6 software, with the window length of 800 bp and the step length of 200 bp.


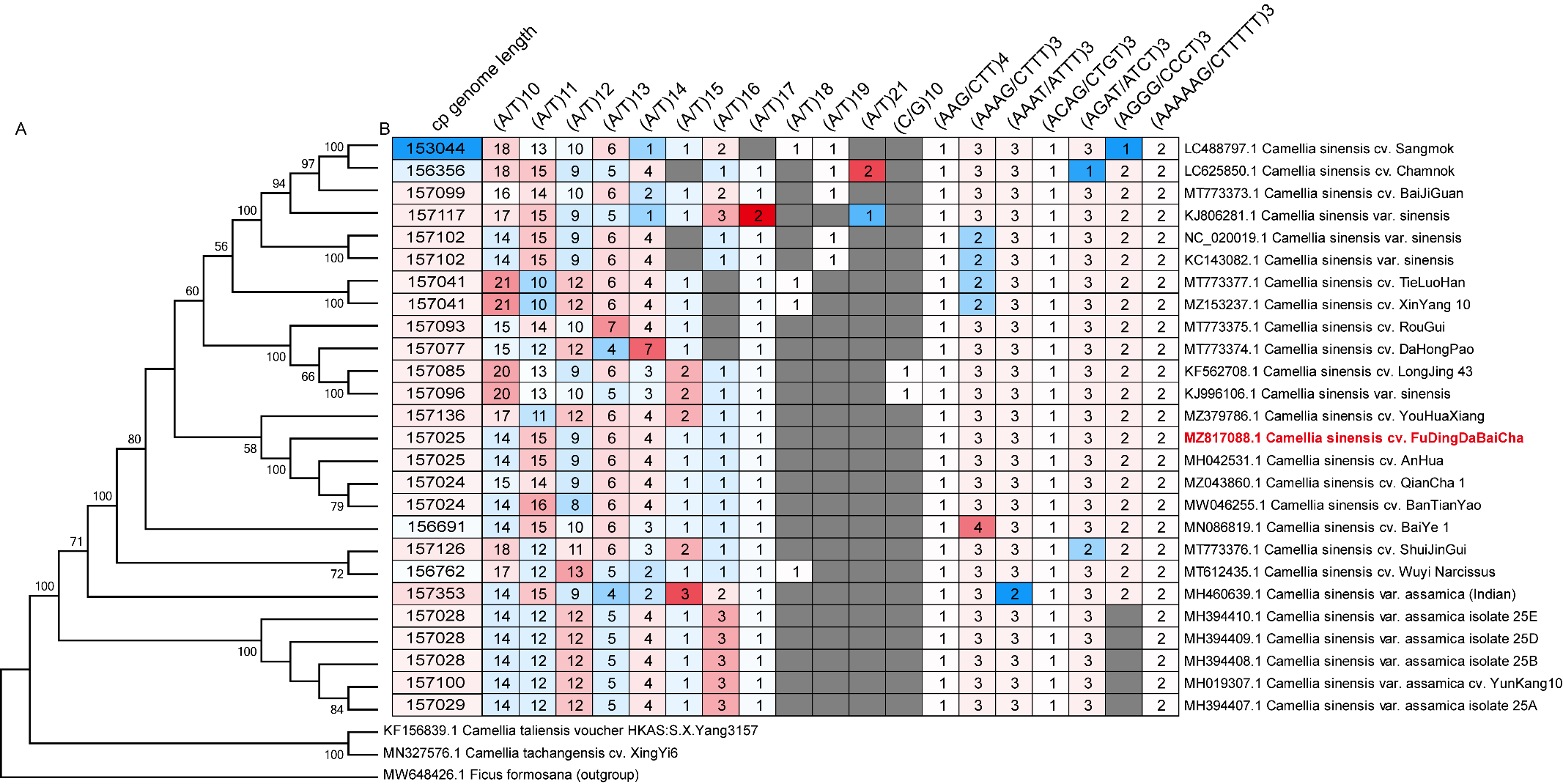


**Figure S4.** Statistics of SSR types and numbers in cp genomes of different tea plant cultivars. The SSR analysis was performed on the cp genomes using MISA tools, with a threshold of 10 for mono-nucleotide, 6 for di-nucleotide, 4 for tri-nucleotide, and 3 for tetra-nucleotide, penta-nucleotide, and hexa-nucleotide SSRs. The minimum distance between two SSRs was set to 100 bp. The heat map was visualized using TBtools software (Chen et al. 2020).


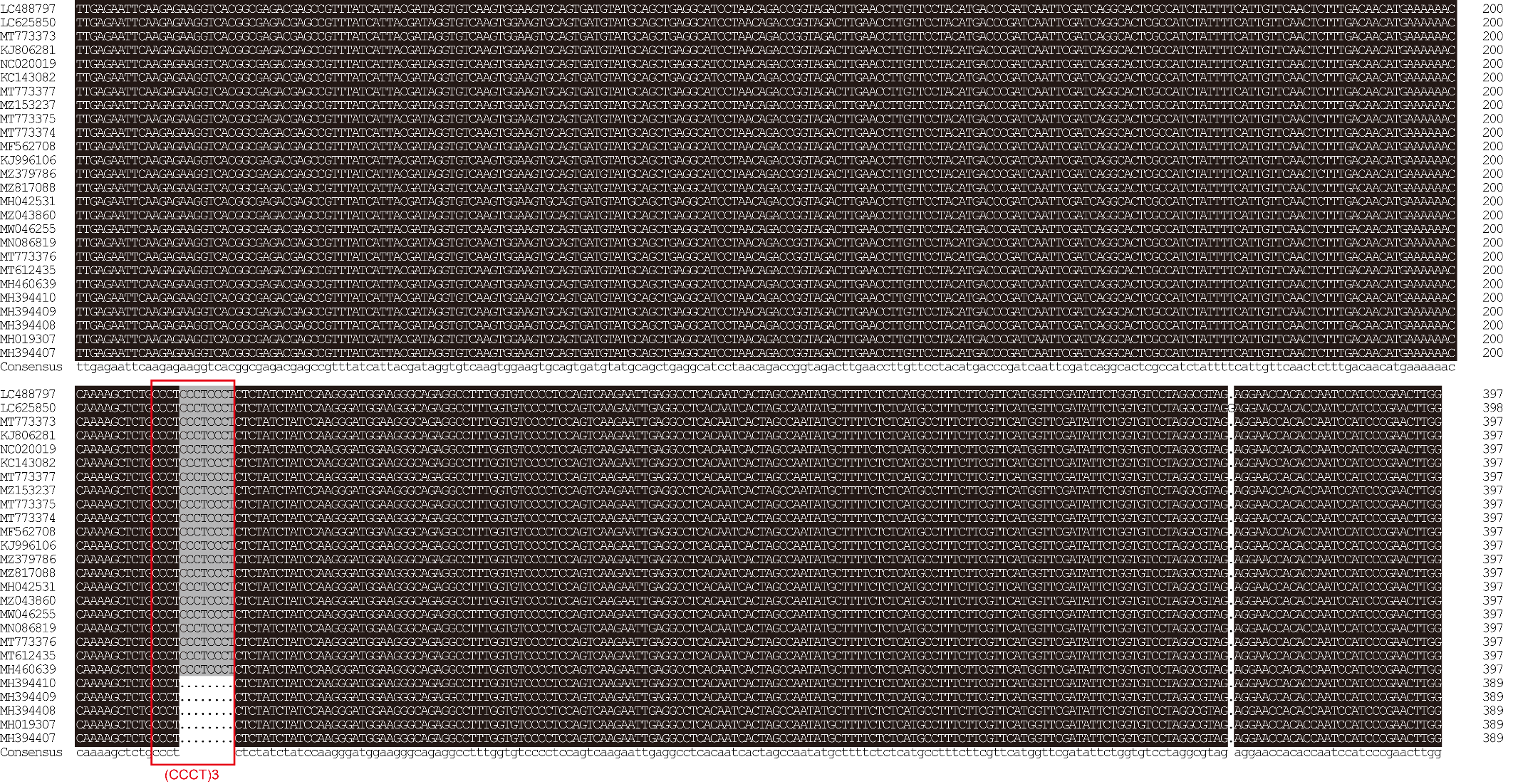


**Figure S5.** A nucleotide sequence based on SSR markers in the cp genome of tea plant that can distinguish *Camellia sinensis* var. *sinensis* (CSS) and *Camellia sinensis* var. *assamica* (CSA) from China.

**Reference**

Chen C, Chen H, Zhang Y, Thomas HR, Frank MH, He Y, Xia R. (2020). TBtools: An integrative toolkit developed for interactive analyses of big biological data. Molecular Plant 13(8):1194-1202.
